# Plumbagin, a Biomolecule with (Anti)Osteoclastic Properties

**DOI:** 10.3390/ijms22052779

**Published:** 2021-03-09

**Authors:** Sevinj Sultanli, Soni Ghumnani, Richa Ashma, Katharina F. Kubatzky

**Affiliations:** 1 Zentrum für Infektiologie, Medizinische Mikrobiologie und Hygiene, Universitätsklinikum Heidelberg, Im Neuenheimer Feld 324, 69120 Heidelberg, Germany; sevinj.sultanli@med.uni-heidelberg.de; 2 Department of Zoology, Savitribai Phule Pune University, Pune 411007, India; soniighumnani04@gmail.com (S.G.); richaashma@unipune.ac.in (R.A.)

**Keywords:** osteoclast, plumbagin, mTOR, translation, ROS, phytotherapy

## Abstract

Plumbagin is a plant-derived naphthoquinone that is widely used in traditional Asian medicine due to its anti-inflammatory and anti-microbial properties. Additionally, plumbagin is cytotoxic for cancer cells due to its ability to trigger reactive oxygen species (ROS) formation and subsequent apoptosis. Since it was reported that plumbagin may inhibit the differentiation of bone resorbing osteoclasts in cancer-related models, we wanted to elucidate whether plumbagin interferes with cytokine-induced osteoclastogenesis. Using C57BL/6 mice, we unexpectedly found that plumbagin treatment enhanced osteoclast formation and that this effect was most pronounced when cells were pre-treated for 24 h with plumbagin before subsequent M-CSF/RANKL stimulation. Plumbagin caused a fast induction of NFATc1 signalling and mTOR-dependent activation of p70S6 kinase which resulted in the initiation of protein translation. In line with this finding, we observed an increase in RANK surface expression after Plumbagin stimulation that enhanced the responsiveness for subsequent RANKL treatment. However, in Balb/c mice and Balb/c-derived RAW264.7 macrophages, these findings could not be corroborated and osteoclastogenesis was inhibited. Our results suggest that the effects of plumbagin depend on the model system used and can therefore either trigger or inhibit osteoclast formation.

## 1. Introduction

Under physiological conditions, bone remodelling is a continuous process that is achieved by the balanced activity of bone-forming osteoblasts and bone-resorbing osteoclasts. Disturbed bone homeostasis can be caused by aging and a reduction in oestrogen or androgen levels, chronic inflammation due to auto-immune pathologies or chronic bacterial infections, or as a consequence of the spread of cancer metastases to the bone environment. The latter is a frequent complication in multiple myeloma, a B cell lymphoma, but more typically found with solid tumours from breast, lung, prostate or thyroid cancers, renal carcinoma, as well as in melanoma, gastrointestinal tumours, and head and neck cancers [1]. Under physiological conditions, the cytokines M-CSF and RANKL are sufficient to differentiate osteoclast precursors from the monocyte/macrophage lineage to osteoclasts. However, tumour-derived factors, such as the proinflammatory cytokines IL-1β, IL-6, TNF-α or IL-17 can enhance osteoclast formation and thus cause osteolysis, i.e., the pathological destruction of bone material [2]. Patients suffer from severe pain as well as fractures, hypercalcaemia and spinal cord compression, all of which can reduce quality of life significantly [3].

Cancer is now the second leading cause of death and despite the enormous progress in therapy options to treat bone cancer, there is still a need for new drugs that are able to overcome resistance of cancer cells that often arises during treatment [4].

In an attempt to find new lead structures, plant-derived molecules, so-called phytochemicals, are studied to develop novel therapeutic tools with decreased cytotoxicity, better drug resistance and high target-specificity. Several phytochemicals show cytotoxicity against cancer cells and are already used clinically, i.e., taxol/paclitaxel from *Taxus brevifolia* that kills tumour cells by inducing multipolar divisions when the cell enters mitosis [5] or vincristine from *Cantharantus roseus* that was approved as the first plant-derived cancer treatment by the FDA [6]. Mechanistically, the effects of phytochemicals range from targeting mitochondrial activity, the induction of ROS production, the inhibition of ABC transporters that confer drug resistance, the inhibition of the proto-oncogene p53 or the induction of programmed cell death through the activation of caspases [4].

Naphthoquinones have a naphthalene-derived structure that is for example used by Vitamin K. The plant-derived naphthoquinone plumbagin (5-hydroxy-2-methyl-1,4-naphthoquinone) is a bioactive compound that was originally isolated from the roots of *Plumbago zeylanica.* Plumbagin is also found in other members of the *Plumbaginaceae* family (*Plumbago capensis*, *Plumbago rosea, Plumbago indica*), as well as in other plants such as the members of *Droseraceae* and *Juglandaceae* families. This naturally occurring naphthoquinone has been in medical use in eastern cultures against infectious diseases, rheumatoid arthritis, dermatological diseases and parasitic infections, respectively, because of its antimicrobial and anti-inflammatory properties. Plumbagin has gained attention in recent cancer studies and encouraging results have been reported in cases of several types of cancer including lung, prostate, breast, cervical, colorectal cancer, leukaemia and melanoma [7]. Each of these investigations of the anti-cancer effects of plumbagin revealed an inhibitory role in multiple mitogenic signalling pathways such as NF-kB, STAT3 and AKT among others [7]. However, to date there has been no study that addresses the detailed mechanism of its activity. Therefore, the signalling molecules known to be targets of plumbagin mostly reflect the central molecules of the respective disease model, but do not give a comprehensive overview on plumbagin-mediated effects. Like other naphthoquinones, plumbagin activates cellular signalling through redox reactions, the inhibition of cellular phosphatases and through direct protein alkylation [8]. In cancer cells, plumbagin mainly acts through the induction of apoptosis via the upregulation of ROS [9,10] and cell cycle arrest through the induction of DNA strand breaks [7,11]. This apoptotic function is supported by other molecular mechanisms that ultimately inhibit tumour growth and metastasis. Due to these encouraging findings, it was also tested whether plumbagin affected cancer-mediated osteoclastogenesis [12,13]. The data suggest that plumbagin helps to improve osteolytic lesions in bone cancer models due to an inhibitory effect on RANKL signalling. The exact mechanism, however, is unclear.

Many investigations that addressed anti-inflammatory effects have been carried out using cancer cell lines or the macrophage cell line RAW264.7. Studies on the effect of plumbagin on M-CSF/RANKL-mediated osteoclastogenesis using primary macrophages from C57BL/6 mice, the most frequently used strain for knock-out models, have not been performed. To better understand the effect of plumbagin on RANKL-mediated differentiation of macrophages into osteoclasts, we used bone marrow-derived macrophages (BMDM) from C57BL/6 mice and stimulation with M-CSF and RANKL, co-stimulation with plumbagin or pre-stimulation with plumbagin before M-CSF/RANKL treatment. Surprisingly, in contrast to the described data, plumbagin did not inhibit the formation of multinucleated, mature osteoclasts. Particularly pre-stimulation with plumbagin showed enhanced osteoclastogenesis compared to M-CSF/RANKL alone. This was accompanied by a fast activation of NF-kB and NFATc1 signalling as well as an upregulated expression of osteoclastic markers. Our data show that plumbagin strongly increases the translational activity of the cell and that pre-incubation resulted in the enhanced surface expression of RANK. We suggest that this increased receptor expression and the general abundance of cellular proteins allow RANKL to signal more efficiently after plumbagin treatment, so that osteoclastogenesis is ultimately enhanced. However, when we performed these experiments using the Balb/c-derived murine macrophage cell line RAW264.7 or osteoclast precursors obtained from Balb/c mice we failed to corroborate the osteoclastic effect of plumbagin. Instead, plumbagin inhibited cell proliferation as well as osteoclast differentiation. The genetic background of mice is known to shape their adaptive and innate immune response [14] and this might explain the dual effect of plumbagin. The ability of plumbagin to exert such divergent activities underlines the need for the further exploration of this molecule. At the same time, the use in an organismal context necessitates the definition of the exact cellular mechanisms involved. Our findings highlight that the use of culture conditions and the genetic background of the animals or the cell lines need to be considered in pharmacological studies as they can drastically affect the outcome and conclusions obtained from experiments.

## 2. Results

### 2.1. Plumbagin Does Not Change Macrophage Marker Molecules

The beneficial effects of plumbagin in inflammation and infection and its ability as a naphthoquinone to induce ROS formation made it an interesting candidate to study its ability to selectively induce apoptosis in cancer cells [7]. Due to the need to improve therapy options for bone-related cancers, it was tested whether the beneficial effect of plumbagin might not only address the tumour itself, but also osteoclast formation. Therefore, in the past ten years, the phytochemical plumbagin was tested for its effect on osteoclastogenesis. 

To study plumbagin-mediated effects in more detail, we set up a model system using bone marrow-derived macrophages from C57BL/6 and RAW264.7 macrophages, respectively. To optimize the experimental conditions, we first tested the toxicity of the compound on bone marrow-derived macrophages. 

**Figure 1:**
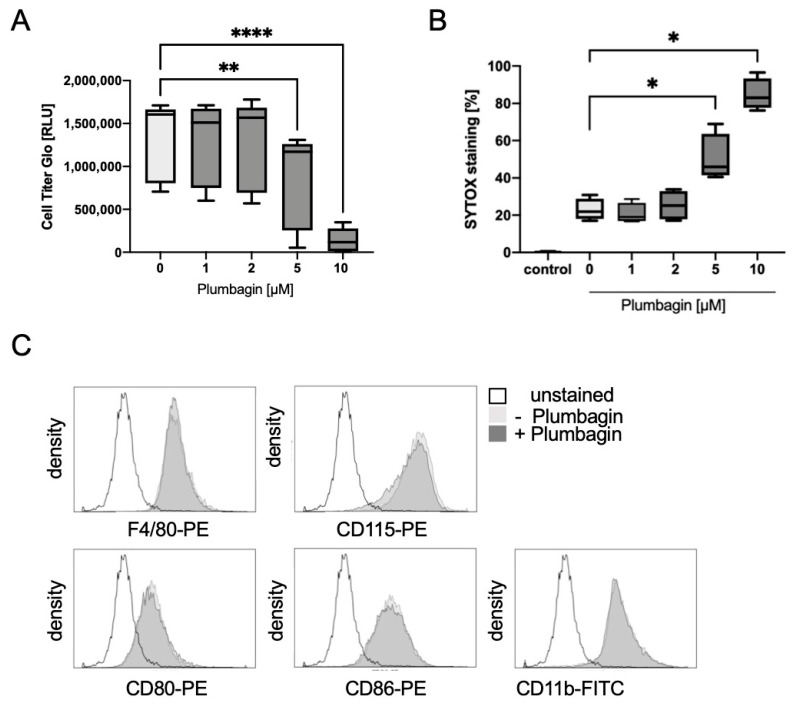
Plumbagin does not change the phenotype of bone marrow-derived macrophages: (**A**) the influence of increasing concentrations of plumbagin in the presence of 25 ng/mL M-CSF was tested after 48 h by measuring cell viability (*n* = 3, with triplicates); (**B**) cell death triggered by increasing plumbagin concentrations was measured after the incubation of the cells with SYTOX after 48 h of treatment in the presence of M-CSF (*n* = 4); statistical analysis was performed comparing the results to the positive control (M-CSF) in (**A**) using Friedman, and in (**B**) using Mann–Whitney test; (**C**) the effect of 2 µM plumbagin was tested on the expression of the macrophage marker molecules F4/80, CD11b, CD80, CD86 and CD115, respectively. The graphs represent one typical example (*n* = 3). *: *p* ≤ 0.05; **: *p* ≤ 0.01; ****: *p* ≤ 0.0001.

For this purpose, we used plumbagin at concentrations of 1, 2, 5 and 10 µM and incubated the cells for 24 h in the presence of the survival factor M-CSF to prevent starvation effects. After lysing the cells, the level of ATP was determined in a chemiluminescent assay. Figure 1A shows that there was no decrease in viability until a concentration of 2 µM, but a significant loss of ATP production at 5 µM and a complete loss of cellular ATP at 10 µM. To complement these data, we determined cell viability by flow cytometry (FACS) analysis using SYTOX green staining to fluorescently label dead cells (Figure 1B). Again, 2 µM plumbagin was the highest concentration of plumbagin that was tolerated well and did not induce cell death. We therefore carried out all further experiments with this concentration. 

Previously, we established bone marrow-derived macrophages (BMDMs) as a suitable model system for differentiation into osteoclasts [15]. Thus, we first determined whether plumbagin treatment would significantly change the phenotype of BMDMs (Figure 1C). We investigated the expression levels of the macrophage markers CD11b, F4/80, CD115 (M-CSF receptor) as well as the activation markers CD80 and CD86. The stimulation of BMDM with plumbagin for 24 h did not change the expression levels and we therefore used these cells to explore the ability of plumbagin to modulate osteoclast differentiation in our further experiments. 

### 2.2. Plumbagin Pre-Treatment Stimulates Osteoclast Formation

In the recent literature, there had been reports that plumbagin might suppress osteoclastogenesis, especially in the context of cancer-related osteolysis [12,13]. However, a thorough investigation of signalling mechanisms is missing from the studies and a number of further plumbagin-related studies have been retracted over time. 

**Figure 2:**
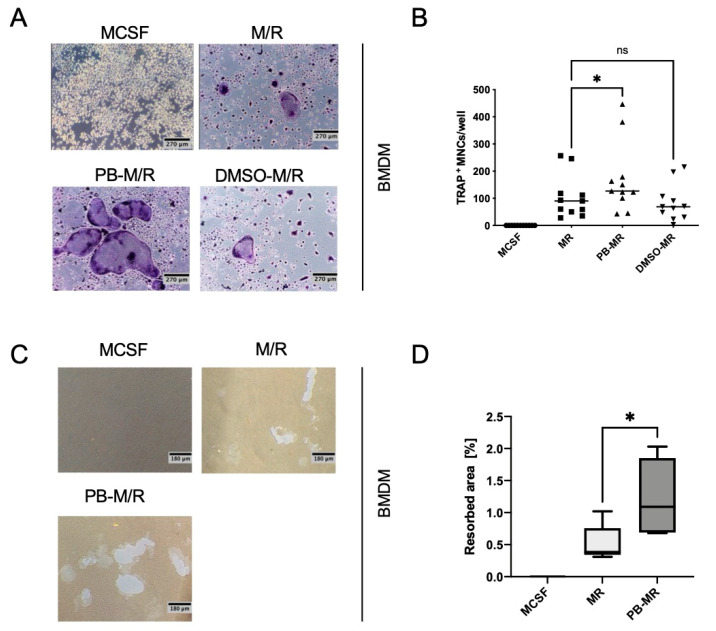
Plumbagin pre-treatment of bone marrow-derived macrophages (BMDMs) facilitates osteoclastogenesis: (**A**,**B**) BMDMs were seeded in a 96-well plate and treated with M-CSF or 2 µM plumbagin on day 0. After 24 h, M-CSF/RANKL was added to the wells of M/R and PB-M/R samples. On day 3, the cells were stimulated with M-CSF, M-CSF/RANKL or plumbagin/M-CSF/RANKL, respectively, until the osteoclasts were fully differentiated; (**A**) shows representative pictures (magnification 100×) and (**B**) presents the quantification of TRAP stains (*n* = 6, five of them with duplicates); (**C**,**D**) BMDMs were stimulated as described in (**A**) on 24-well osteo assay surface plates before quantifying the resorbed area on day 10 (*n* = 3, with duplicates); statistical analysis was carried out comparing the results to the positive control (M-CSF/RANKL) using Friedman test in (**B**) using a Friedman test and a Mann–Whitney test in (**D**). *: *p* ≤ 0.05; ns: *p* > 0.05.

To understand the effect of plumbagin on osteoclast formation on the signalling level, we stimulated macrophages with 2 µM plumbagin and the cytokines M-CSF and RANKL simultaneously (M/R+PB). To our surprise, plumbagin (PB) did not change the ability of macrophages to differentiate into osteoclasts (Figure S1A,B) in our model system. Therefore, we decided to pre-incubate the cells for 24 h with plumbagin before the addition of M-CSF and RANKL (PB-M/R). On day 3, the cells were re-stimulated with plumbagin and M-CSF/RANKL or cytokines only in control cells (Figure S1C). In addition, a solvent control was included to prevent any unwanted effect of DMSO on cell viability or differentiation. Again, there was no inhibition of osteoclast formation compared to the positive control that had been stimulated with M-CSF/RANKL only (Figure 2A,B). Instead, the quantitative evaluation of the performed assays for expression of the osteoclast specific tartrate resistant acidic phosphatase (TRAP) showed that there was a statistically significant increase in osteoclast numbers and size (Figure 2A,B). The effects of the solvent DMSO could be excluded as the differentiation of control cells was comparable to M-CSF/RANKL treated cells. 

To test whether the obtained osteoclasts also showed comparable levels of resorptive activity, the cells were seeded on osteoplates and treated as described above (Figure 2C,D). Again, the data clearly show an increase in the resorbed area due to the enhanced number of osteoclasts in the pre-treated group. 

### 2.3. Activation of Intracellular Signalling Pathways

Although a number of proteins have been described to be inhibited after plumbagin treatment, it is yet unclear how plumbagin interferes with intracellular signalling, as there is no target molecule or mechanism of activation known. Since the 24 h pre-stimulation with plumbagin affected the cellular plasticity towards a more osteoclastogenic phenotype, we wanted to investigate the reason for this increased susceptibility towards M-CSF/RANKL treatment. 

**Figure 3:**
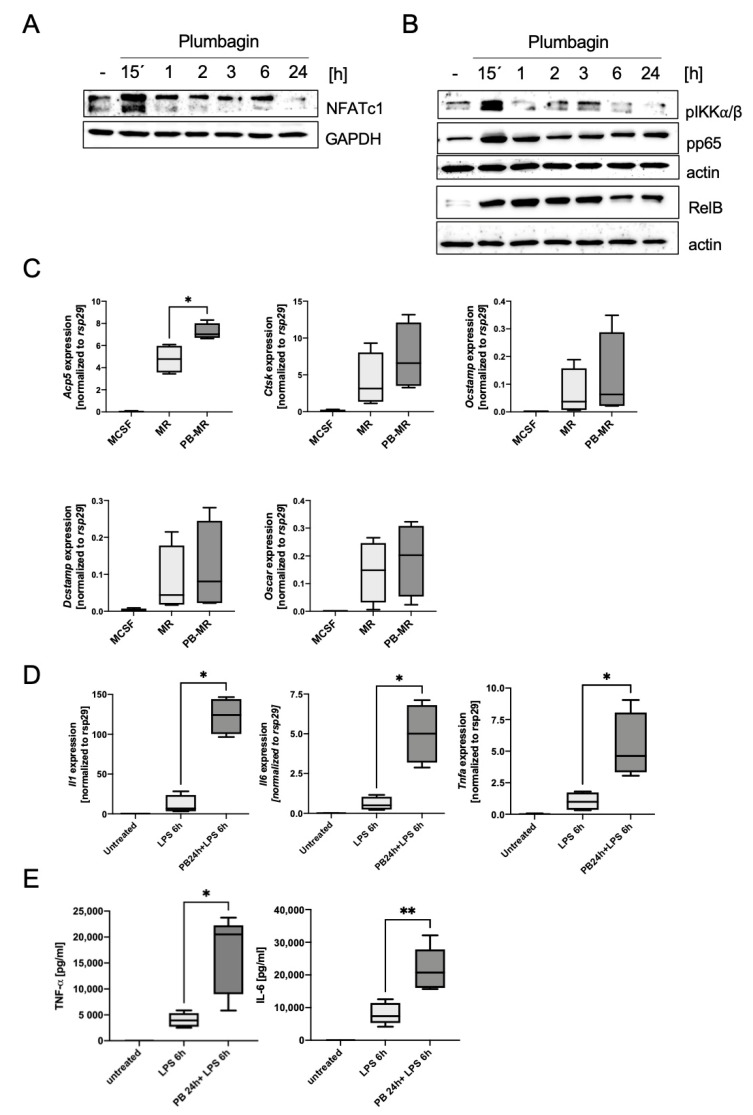
Plumbagin induces the rapid activation of cellular signalling in BMDMs: (**A**,**B**) Western blot analysis to test typical osteoclast signalling molecules was performed using antibodies against NFATc1 (**A**) and molecules of the NF-κB pathway (**B**). GAPDH and actin were used as loading controls. The blots are typical examples out of three independent experiments; (**C**) differentiating osteoclasts were analysed on day 3 by RT-PCR to determine the expression of the osteoclastic marker genes *Acp5, Ctsk, Ocstamp, Dcstamp and Oscar*, respectively (*n* = 4). Statistical analysis comparing the results to the positive control (M-CSF/RANKL) was performed using Mann–Whitney test; (**D**,**E**) BMDM were either pre-stimulated with plumbagin for 24 h before stimulation with 100 ng/mL LPS for 6 h or stimulated directly with LPS. (**D**) RT-PCR was performed to determine the expression of the osteoclastogenic, pro-inflammatory cytokine genes *Il6, Il1 and Tnfa*, respectively (*n* = 4); (**E**) the supernatants of the samples were subjected to ELISA to determine the levels of produced cytokines (*n* = 5). Statistical analysis comparing the results to the positive control (LPS) was carried out using a Mann–Whitney test in (**D**) and (**E**). *: *p* ≤ 0.05; **: *p* ≤ 0.01.

We therefore incubated the cells with plumbagin for various time-points ranging from 15 min to 24 h and investigated its effect on proteins activating osteoclast differentiation. Interestingly, we found that the expression levels NFATc1, the master transcription factor of osteoclastogenesis, were most strongly increased already after 15 min. Changes in the expression pattern could be observed until at least 6 h compared to the untreated control samples (Figure 3A). In our subsequent analysis of activation of the NF-κB pathway, we determined the serine phosphorylation levels of pIKKα/β and p65 (Figure 3B). Again, we observed a significant change in the phosphorylation levels that was most pronounced at early time-points and suggested the activation of this second pathway that is important for osteoclast differentiation. In addition, we observed an increase in the expression of the non-canonical NF-κB member RelB that is central in mitochondrial biogenesis during differentiation [16]. These data suggest that an early plumbagin-mediated signalling event is able to change the fate of the cell and that constitutive activation of signalling events is not involved. Figure 3C shows that the activation of these pathways indeed has an impact on the expression of osteoclast-specific genes. *Acp5* (TRAP), *Ctsk* (Cathepsin K), *Ocstamp*, *Dcstamp* and the osteoclast associated receptor *Oscar* are reproducibly upregulated in the samples pre-treated with plumbagin (PB-M/R) compared to M-CSF/RANKL or M-CSF-treated control cells. The direct comparison of p65 phosphorylation and NFATc1 expression between M-CSF/RANKL-treated cells, plumbagin pre-stimulated cells and cells simultaneously stimulated with plumbagin and M-CSF/RANKL showed that plumbagin increases the activation of these pathways compared to M-CSF/RANKL (Figure S2A).

Since plumbagin strongly activated p65, we wanted to investigate whether this would cause the production of proinflammatory cytokines such as IL-1β, IL-6 or TNF-α. In addition to their pro-inflammatory function, these cytokines can also act as inducers of osteoclastogenesis, which might provide a potential mechanism of osteoclast differentiation [17]. When we performed the RT-PCR analysis of plumbagin and lipopolysaccharide (LPS)-treated cells, plumbagin alone did not induce cytokine expression (Figure S2B). Therefore, we wanted to see if plumbagin could change the responsiveness of the cells towards the stimulation with the TLR4 ligand LPS, similarly to the increase in osteoclastogenesis. Therefore, we pre-treated BMDMs for 24 h with plumbagin before adding LPS (100 ng/mL) for an additional 6 h period. Our data show that induction of *Il6*, *Il1b* and *Tnfa* in these cells was enhanced. This could be corroborated by ELISA assays of cytokines secreted into the supernatant (Figure 3D,E). Secretion of IL-1β was not observed due to the absence of a second inflammasome activation signal. 

In summary, our data show that plumbagin rapidly induces cellular signalling events once it entered the cell and that osteoclast-related pathways such as the NFATc1 and NF-kB signalling are activated, although pro-inflammatory cytokines are not produced and cannot be the reason for the enhanced osteoclast formation. However, it remained unclear why such an early induction in signalling could lead to a long-lasting effect of osteoclast differentiation.

### 2.4. Plumbagin Inhibits Osteoclastogenesis in RAW264.7 Macrophages

**Figure 4:**
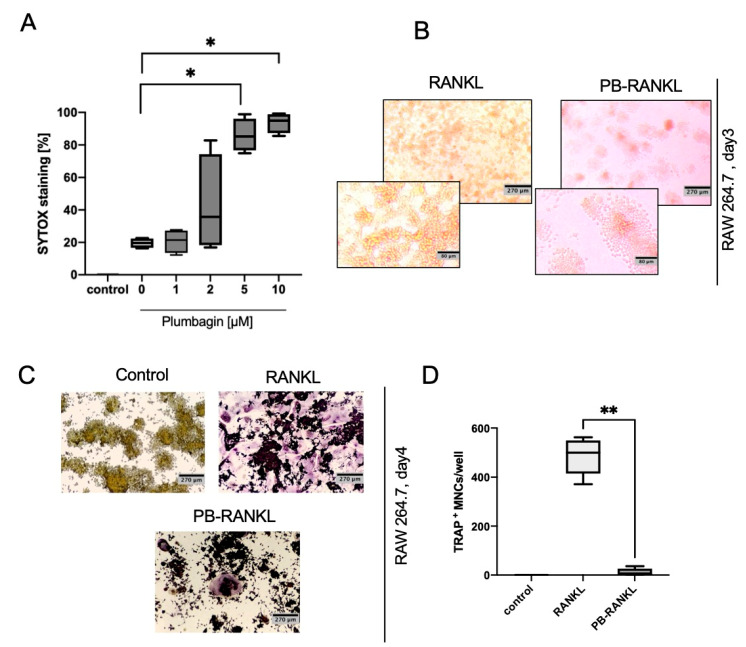
Plumbagin inhibits osteoclastogenesis in RAW264.7 macrophages. (**A**) RAW264.7 cells were treated with increasing concentrations of plumbagin for 24 h and cell viability was measured after incubation with SYTOX. Statistical analysis was performed comparing the results to the control in using a Mann–Whitney test (*n* = 4); (**B**,**C**) and (**D**) RAW264.7 cells were seeded in a 24 well plate and treated with RANKL or plumbagin (1 µM) /RANKL. On day 3, cells were restimulated with RANKL, plumbagin/RANKL, respectively, until the osteoclasts were fully differentiated; (**B**) shows representative pictures and (**C**) the quantification of TRAP stains (*n* = 3, with duplicates); (**D**) one day before the TRAP staining was performed, light microscopic pictures (magnification 100×) were taken to document cell viability and cell numbers. *: *p* ≤ 0.05; **: *p* ≤ 0.01.

To understand why there was such a discrepancy between our data and the published effects of plumbagin on osteoclastogenesis that had postulated an inhibitory effect, we decided to use the mouse macrophage cell line RAW264.7 which had served as a model system for many other studies. To determine the viability of RAW264.7 macrophages in response to plumbagin, we performed a SYTOX assay. In contrast to BMDMs which tolerated plumbagin well at a 2 µM concentration (Figure 1B), we already observed an increase in the number of dead cells at 2 µM that was more pronounced and became statistically significant at 5 and 10 µM (Figure 4A). Therefore, osteoclast differentiation was performed at 1 µM plumbagin and the number of TRAP-positive cells was determined on day 4 of differentiation (Figure 4B,C). In contrast to our previous results, we observed a significant inhibition of osteoclastogenesis after the pre-treatment of cells with plumbagin. Because TRAP staining does not allow the analysis of cell viability, we documented cellular viability by light microscopic analysis prior to TRAP analysis. It could be observed that although viability was not impaired, 1 µM of plumbagin prohibited cell proliferation and that the number of cells as well as the metabolic consumption of the medium was decreased (Figure 4D). In vitro studies using Balb/c mice also showed that osteoclastogenesis is inhibited (Ghumnani et al., 2021 manuscript under preparation). Here, osteoclast-specific genes were significantly less expressed in the presence of plumbagin, which seems to be caused by an inhibition of NFATc1 nuclear translocation (Figure S3A,B). 

### 2.5. Mitochondrial Activity

Plumbagin has been described to exert a strong impact on the formation of ROS which is part of its anti-tumour effects [7]. While ROS is detrimental to cellular integrity, especially for cancer cells that are more likely to undergo apoptosis in the presence of ROS, ROS can also act as a secondary messenger that triggers osteoclastogenesis [18]. 

**Figure 5:**
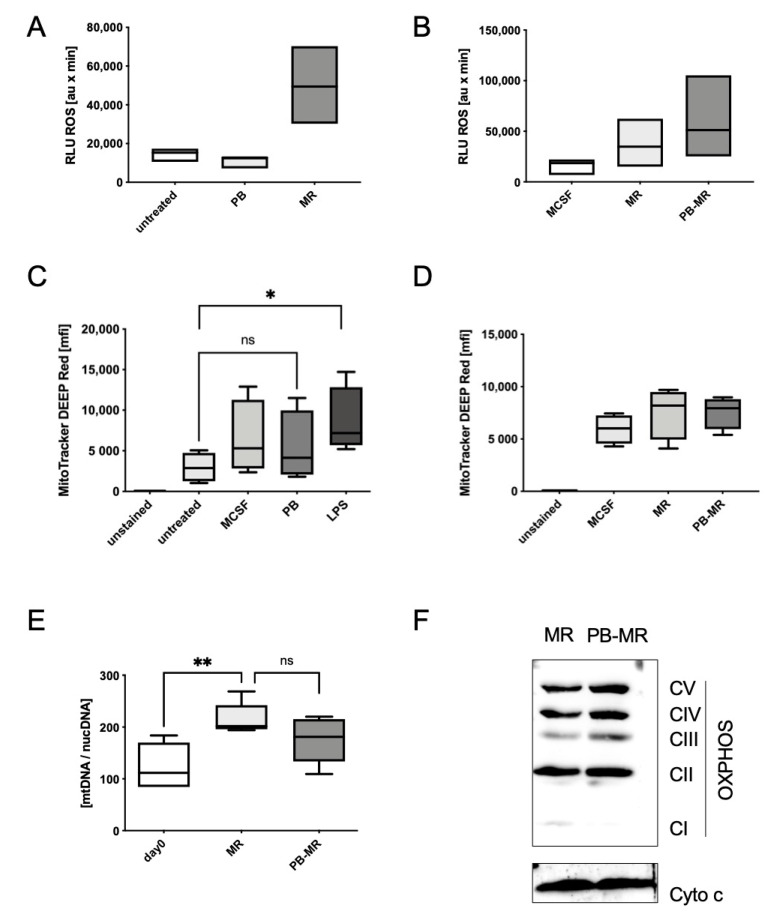
Plumbagin impacts ROS production in the presence of M-CSF/RANKL in BMDMs: (**A**) cells were incubated for 48 h with plumbagin or treated with M-CSF/RANKL before ROS production was analysed in a (iso)luminol-based assay (*n* = 3); (**B**) cells were incubated for 24 h with M-CSF/RANKL, plumbagin or pre-treated with plumbagin before the addition of M-CSF/RANKL and ROS production was analysed in a (iso)luminol based assay on day 2 (*n* = 3); (**C**) BMDMs were incubated for 6 h, as indicated. Mitochondrial activity was measured using Mitotracker deep red stain for 30 min before FACS analysis (*n* = 4); (**D**) BMDMs were pre-treated for 24 h with plumbagin before the addition of M-CSF/RANKL, or treated with only M-CSF and M-CSF/RANKL, respectively. Mitochondrial activity was measured using Mitotracker deep red stain for 30 min before FACS analysis (*n* = 4); (**E**) BMDMs were differentiated as indicated and the mitochondrial copy number was determined by comparing mitochondrial and nuclear DNA levels (*n* = 5); (**F**) the expression of OxPhos components was analysed in the mitochondrial extracts from cells treated with M-CSF/RANKL or plumbagin pre-stimulated (PB-M/R) cells by Western blot analysis on day 3. The blot is one typical example out of 3 experiments. Statistical analysis was performed comparing the results to the untreated control for (**C**) and for (**F**) to the day 0 or positive control (M-CSF/RANKL) using a Mann–Whitney test. *: *p* ≤ 0.05; **: *p* ≤ 0.01. ns. *p* > 0.05.

Therefore, we then addressed whether an upregulation of ROS formation could be the reason for the observed increase in osteoclastogenesis. While there was no ROS production after plumbagin alone (Figure 5A), a slight increase in ROS levels for cells pre-treated with plumbagin before M-CSF/RANKL stimulation could be detected using a luminol assay (Figure 5B). This suggests that plumbagin is able to exacerbate RANKL-mediated ROS production. Therefore, we then investigated if we could detect an impact of plumbagin on mitochondrial activity, the number of mitochondria or the expression of OxPhos components, respectively, since all of these factors are known to contribute to osteoclast formation and activity [19]. In contrast to the ROS assay in Figure 5B, which measured the total ROS levels from mitochondrial and cytoplasmic ROS sources, a Mitotracker deep red stain for activated ROS-producing mitochondria did not detect an increase in mitochondrial activity after 6 h of incubation with plumbagin compared to M-CSF-treated cells (Figure 5C). 

Similar results were obtained when we used this assay to compare M-CSF with M-CSF/RANKL and plumbagin pre-treated M-CSF/RANKL cells (Figure 5D). When we compared mitochondrial copy numbers between M-CSF/RANKL and plumbagin pre-treated cells, a small and statistically not significant decrease could be observed in plumbagin pre-treated osteoclasts (Figure 5E). When we investigated the expression levels of ETC complex proteins (Figure 5F), we noticed a slight but reproducible increase in the expression of complexes III–V when M-CSF/RANKL treatment was preceded by plumbagin incubation. However, since all of these effects remained subtle, we reasoned that the observed increase in ROS formation is not involved in priming the cells towards increased M-CSF/RANKL susceptibility through plumbagin pre-incubation. Rather, we hypothesised that the increase in ROS occurs as a consequence of pre-treatment with plumbagin due to enhanced cellular activity and the observed increase in osteoclastogenesis. 

### 2.6. Plumbagin Triggers Protein Translation

Because cellular differentiation relies on the ability of cells to create building blocks and to make use of anabolic pathways, we investigated the ability of plumbagin to influence the activation of key enzymes involved in the regulation of metabolic activity. Adenosine monophosphate-activated protein kinase (AMPK) is a sensor of cellular ATP levels. When ATP is depleted from the cells after excess anabolic activities, AMPK becomes activated through phosphorylation on T172 [20]. Phosphorylated AMPK can then switch the cell from anabolic processes towards the generation of ATP through the activation of the TCA cycle. As a marker for anabolic processes, we used the mTOR effector molecules p70S6K and 4E-BP1, as both of these proteins are regulators of protein translation [21]. 

**Figure 6:**
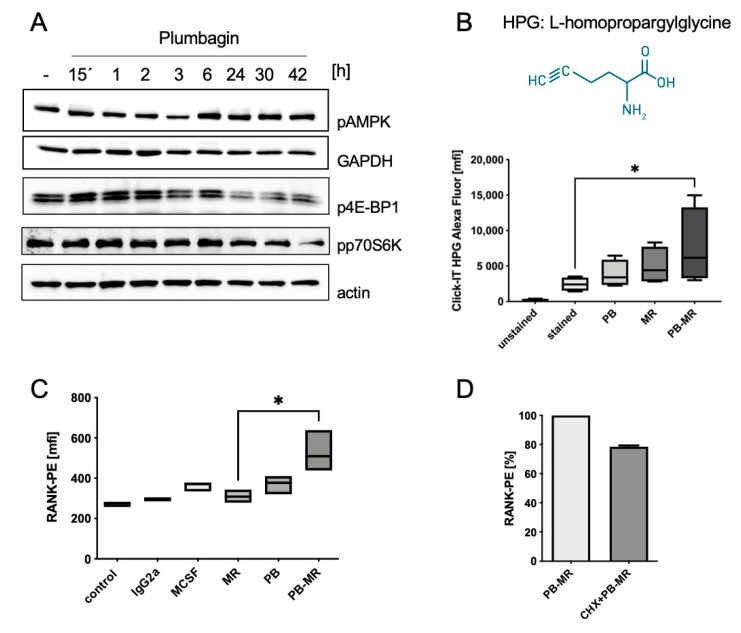
Plumbagin pre-treatment of BMDM facilitates osteoclastogenesis: (**A**) the expression of the metabolic signalling molecules pAMPK, p4E-BP1 and p70S6K was analysed from whole cell lysates at the indicated time-points of plumbagin stimulation. The blots show one typical example out of 3 experiments; (**B**) translational activity was measured using a Click-IT assay and the subsequent measurement of the incorporated, Alexa488 click-labelled HPG-containing proteins. Cells were treated for 6 h as indicated before chasing for 30 min with HPG. Untreated unstained and untreated stained cells were used as negative controls for background fluorescence and background translational activity, respectively. Fixed cells and Alexa488 were then measured by FACS analysis (*n* = 4). Statistical analysis was performed by comparing results to the untreated control using a Friedman test; (**C**) RANK expression was measured by FACS analysis using a phycoerythrin (PE)-labelled antibody; (**D**) cells were pre-treated with plumbagin or 10 µM cycloheximide to block protein translation (*n* = 3). Statistical analysis was implemented by comparing the results to the positive control (M-CSF/RANKL) using Kruskal–Wallis test. * *p* > 0.05.

While we did not observe major changes in pAMPK levels at early timepoints (Figure 6A), there seemed to be in increase over time (>6 h). Much to the contrary, 4EB-P1 phosphorylation increased early (15 min) and decreased after 6 h of stimulation. A similar pattern was observed for p70S6K, although the initial increase was observed after 1 h of plumbagin treatment. Again, a significant drop in protein phosphorylation that coincided with the upregulation of pAMPK could be observed at time-points later than 24 h. Since p4E-BP1 and p70S6K both play a role in the initiation of protein translation, we next checked if we could detect translational activity in the cells. To this end, we performed a fluorescence-based pulse-chase experiment and the cells were grown in methionine-free medium before L-homopropargylglycine (HPG), a reactive methionine analogue, was added for 30 min. The addition of CuSO_4_ and Alexa488 after 6 h started a click reaction and coupled the dye to incorporated HPG residues. FITC fluorescence was then measured and analysed using a flow cytometer. The data shown in Figure 6B clearly reveal an increase in translational activity in the presence of plumbagin alone with no other stimuli, even though the effect of M-CSF/RANKL treatment is more pronounced. This can probably be explained by the fact that plumbagin treatment was performed in the absence of any other survival factors such as M-CSF. As a control, the cells were stimulated with plumbagin and the translation inhibitor cycloheximide (Figure S4). However, pre-treatment with plumbagin was able to synergistically increase the translational activity of the cells and resulted in a significant upregulation (approximately 45%) compared to M-CSF/RANKL alone. 

As the translation assay could only show that protein translation is upregulated in general but did not give information on the exact proteins that were translated, we decided to specifically investigate whether the expression of RANK, the receptor for RANKL, was among the translated proteins (Figure 6C). The FACS analysis of the receptor surface expression showed an increase in response to M-CSF as the main regulator of RANK expression [22] as well as to plumbagin treatment. However, the expression of RANK was much higher after pre-treatment with plumbagin and the subsequent addition of M-CSF/RANKL. These data therefore correspond very well to the synergistic effect observed for the translational assay in Figure 6B. As a consequence, we hypothesize that plumbagin pre-treated cells would be more susceptible to the following M-CSF/RANKL treatment and thus differentiate more efficiently into osteoclasts. To prove that the increase in RANK expression was dependent on protein translation and not a change in protein stability, we co-incubated plumbagin-treated cells with cycloheximide to inhibit protein synthesis. Indeed, Figure 6D shows that cycloheximide treatment led to a 20% reduction in RANK expression compared to plumbagin pre-treated osteoclasts. This result suggests that the observed increase in RANK cell surface localisation after plumbagin treatment was at least partially caused by enhanced translation. 

## 3. Discussion

Plant-derived phytochemicals contain active substances that can be exploited pharmacologically. These chemicals are often secondary metabolites that are generated by the plant to defend itself against external challenges [23]. Phytotherapy aims to exploit the potential of such drugs as alternative treatment options in cancer and other non-communicable diseases. Several phytochemicals are successfully being used in the clinical context, showing the relevance of this approach. The plant-derived naphthoquinone plumbagin is a bioactive compound that was originally isolated from the roots of the *Plumbago* genus [24]. Because plumbagin is an effective mediator of ROS production in a variety of different cells, the focus of research has been on its anti-cancer properties [10]. The first report that addressed the modulatory abilities of plumbagin on macrophages was published in 1995 and suggested a potentiation of bactericidal activity [25]. Balb/c mice have been fed with plumbagin for 6 weeks, before peritoneal macrophages were isolated and subsequently infected with *Staphylococcus aureus*. The data showed a dose-dependent increase in bacterial killing in response to the plumbagin supplementation of the food. While the paper could confirm a significantly higher release of various ROS, it was not investigated if this was supported by a concomitant increase in NF-kB activity and cytokine production. Although our own data do not provide evidence for a direct effect of plumbagin on ROS production, plumbagin was still able to moderately increase ROS levels in the presence of the ROS inducer M-CSF/RANKL (Figure 3A,B). Indeed, the ability to produce ROS can be expected to be different between cells. Cancer cells already show a predisposition to produce more ROS, which ultimately renders them more sensitive towards additional ROS [26]. While innate immune cells are potent producers of ROS as part of their microbial killing mechanisms, differences between the cells can still be found and granulocytes or peritoneal macrophages are more likely to produce ROS than BMDMs [27,28].

Studies that investigated the effect of plumbagin on cytokine production describe a downregulation of NF-kB activation and a decrease in the release of pro-inflammatory cytokines [29,30,31,32,33]. To our surprise, we found that plumbagin rapidly induces the phosphorylation of p65. In addition, our own data convincingly show that plumbagin prestimulation enhances LPS-mediated NF-kB activation and increases the production of IL-6, IL-1β and TNF-α. The increased production of cytokines fits well with our finding of an increase in p65 activation in response to plumbagin treatment that might cause an enhanced ability of the cells to react to further stimuli such as LPS or M-CSF/RANKL that both make use of the same pathway. Indeed, it would be astonishing for an inflammatory model if ROS levels increased while the production of cytokines would decrease. As mitochondrial ROS levels and the expression of pro-inflammatory cytokines are coupled [34], it can be expected that innate immune cells that produce increased levels of ROS would also increase cytokine production. A possible explanation is that the co-stimulation of plumbagin with high doses of LPS (1 µg/mL) in combination could reduce cell viability, e.g., through an increase in ROS, and would thus cause a reduction in inflammation [29,31,32]. Zhang et al. used a Balb/c macrophage model that was the closest to our own C57BL/6 model system to investigate the effect of plumbagin on immunometabolism. Again, it was claimed that plumbagin inhibits IL-1β production, however, supernatants were measured after 2 h of stimulation, which is different from the 24 h of pre-stimulation and 6 h of LPS stimulation used in this manuscript. The authors observe an increase in the oxygen consumption rate of the BDMDs, while the extracellular acidification rate, a sign of aerobic glycolysis, was reduced [33]. Interestingly, we could observe that plumbagin has the ability to increase the expression of the electron transport chain proteins during osteoclastogenesis. It is possible that this also plays a role during inflammation. Still, our data show a pronounced early increase in mTOR-mediated anabolic pathways and protein translation as we found them during glycolysis under inflammatory conditions. mTOR also plays a central role in osteoclastogenesis under physiological and pathological conditions [35,36]. Our data show that protein translation is increased through plumbagin and we have evidence that RANK is among these proteins. The increase in RANK surface localisation is measurable without any other cytokine present that would sustain cell survival and thus must be quite strong. The effect is therefore most pronounced when cells are treated with plumbagin and M-CSF/RANKL. Although RANKL and M-CSF are usually described as the critical factors that determine if osteoclast differentiation takes place, the expression of the receptor RANK is also of importance. A couple of recent publications highlight the fact that the regulation of RANK expression is an effective means to change the effectiveness of osteoclast differentiation [37,38,39]. Indeed, RANK expression on macrophages is quite low and it is therefore not surprising that RANK abundance can be a limiting factor in osteoclastogenesis. For example, di Ceglie et al. recently (2019) described that the endogenous DAMP and marker for sterile inflammation in rheumatoid arthritis, the alarmin S100A9, reduced M-CSF-induced RANK expression to limit osteoclastogenesis [39]. 

At this point, we do not know how signalling pathways are activated by plumbagin and what the central regulator of plumbagin-transduced signals is, as we specifically looked for signalling molecules that are important during osteoclastogenesis. Using the swiss target prediction tool, we verified some of the already published targets [40], but more work is needed to understand how plumbagin activates specific signalling events in these cells. While there are numerous experimental set-ups that looked at signalling molecules that might be relevant for their respective experimental system, a comprehensive study that identifies the mode of activation is missing. Predictive software suggests a number of quite different molecules; however, the predictions are based on a similarity search and ultimately rely on the experimental data that have been obtained for plumbagin or similar naphthoquinones. Klotz et al. suggest a variety of possible scenarios regarding naphthoquinone-mediated signalling: (a) the activation of RTK and subsequent downstream signalling; (b) the inhibition of phosphatases and the resulting increase in protein phosphorylation and the subsequent activation of downstream molecules; (c) the direct redox-mediated activity change of molecules; as well as (d) the alkylation of proteins [8]. Our data suggest that the activation of signalling pathways occurs rapidly, i.e., within minutes and that a strong increase in protein tyrosine phosphorylation goes along with it (data not shown). This suggests that it is possible that specific kinases are activated or phosphatases inhibited. PTP1B, for example, is an important protein tyrosine phosphatase in haematopoietic cells and macrophages and CD45-deficient mice have a defect in osteoclast formation due to reduced cellular motility. Of note, the inhibition of phosphatases is frequently used in osteoporosis therapy with alendronate [41]. Clearly, further studies are needed for plumbagin to assess its suggested usefulness for therapeutic purposes.

Currently, there are two other reports on the effect of plumbagin on osteoclast formation in the context of breast cancer [12,13] that both suggest an inhibitory effect of plumbagin on osteoclastogenesis. We can corroborate this negative impact on osteoclast formation found by Sung et al. who used a RAW264.6 model [13]. Li et al. directly cultured primary bone marrow cells from C57BL/6 mice with plumbagin that, compared to bone marrow-derived macrophages, might show a different tolerance towards the effects of plumbagin. In contrast, when we studied the effect of plumbagin on C57BL/6 BMDM, we did not see any difference in the ability to differentiate into osteoclasts between M-CSF/RANKL and plumbagin/M-CSF/RANKL co-cultured cells (Figure S1A,B). Instead, pre-treatment even enhanced the differentiation process. Because RAW264.7 macrophages are derived from Balb/c mice, we hypothesize that the observed differential effects of plumbagin could be dependent on the genetic background of the mice. It has been known for a long time that these mice act differently in the context of an infection. In mice infected with Leishmania, it was found that Balb/c mice had a strong Th2 response while C57BL/6 mice primarily exerted a Th1 phenotype response [14,42]. Our data clearly indicate that the differences between experimental systems and the reproducibility of data may need to be discussed in the context of differential immune activation, especially in the context of pharmacological treatment.

## 4. Materials and Methods

### 4.1. Mice

C57BL/6 wild-type mice were purchased from Janvier Labs (Le Genest St. Isle, France). Mice were maintained under specific pathogen-free (SPF) conditions in compliance with the German policies on animal welfare (approval number: T-61/18, 10/2018).

### 4.2. Reagents

Tissue culture reagents were purchased from Anprotec (Bruckberg, Germany), Biochrom GmbH (Berlin, Germany), PAN biotech (Aidenbach, Germany), Thermo Scientific (Langenselbold, Germany), Merck and Sigma. Antibodies against phosphorylated NF-kB, AMPKα (Thr172), 4E-BP1 (Thr37/46), p70 S6 Kinase (Thr 389), Tyr-100, IKKα/β and total NFATc1 as well as RelB were purchased from Cell Signaling Technology (Leiden, The Netherlands). Antibodies against GAPDH, β-actin and cytochrome C were obtained from Proteintech (Manchester, UK). OXPHOS antibody cocktail was purchased from abcam (Cambridge, UK). Secondary horseradish peroxidase (HRP)-linked antibodies were obtained from Cell Signaling Technology (anti-rabbit IgG, anti-Mouse IgG). PE-conjugated anti-F4/80, PE-conjugated anti-CD86, PE-conjugated CD-115, CD265 (RANK), and PE- conjugated IgG2a were provided by BioLegend (San Diego, CA, USA). PE-conjugated anti-CD80 was purchased from BD Biosciences (Heidelberg, Germany). SYTOX Green was procured from Thermo Scientific (Langenselbold, Germany), and MitoTracker DEEP Red FM stain from Cell Signaling Technology (Leiden, The Netherlands). PCR primers were purchased from Apara (Denzlingen, Germany) or Biomers (Ulm, Germany).

### 4.3. Differentiation of BMDMs

Bone marrow (BM) cells were isolated from the femur, tibia and humerus 12-20-week-old C57BL/6 female mice. To generate BMDMs, BMC were treated with L929-cell conditioned medium (LCCM) as described previously [15]. On day 1, BM cells were resuspended in 20 mL of DMEM medium. On day 4, the cells were restimulated with 30% LCCM and incubated for an additional 3 days.

### 4.4. Stimulation

Cells were stimulated with 2 µM plumbagin (Cayman Chemical, Ann Arbor, USA, purity ≥98%) or 50 ng/mL rec. mouse sRANKL, 25 ng/mL rec. mouse M-CSF (all from Biotechne, Abington, UK). Plumbagin was used as 53 mM stock dissolved in DMSO and diluted with medium to a final concentration of 1–10 µM. LPS was from *Salmonella enterica* serotype abortus equi (L5886 Sigma, Taufkirchen, Germany) and used at 100 ng/mL.

### 4.5. TRAP Staining

A total of 5 × 10^4^ cells were seeded in 200 µL of medium in 96-well plates and treated as described. Cells were then fixed and stained using Acid Phosphatase, Leukocyte (TRAP) Kit (Sigma-Aldrich, Taufkirchen, Germany). TRAP-positive cells with three or more nuclei were counted as osteoclasts.

### 4.6. Bone Resorption Assay

A total of 2 × 10^6^ cells were seeded in 1 mL of medium in Osteo Assay Surface 24-well plates (Corning, NY, USA) for 10 days and stimulated as indicated. On day 10, cells were washed with PBS and incubated in 250 µL sodium–hypochlorite (5–7%) for 5 min at RT. Wells were washed twice with H_2_O and left to dry overnight. The resorbed area was measured with ImageJ software.

### 4.7. Cell Viability Assay

As indicated, 5 × 10^4^ cells per well were seeded in a translucent 96 well microplate and stimulated with plumbagin. To quantify ATP levels, the supernatant was removed and the CellTiter-Glo Reagent (Promega, Mannheim, Germany) was added according to the manufacturer’s protocol. Cell lysates were transferred to white 96-well assay plates and measured with a LUMIstar Optima (BMG, Offenburg, Germany) luminometer.

### 4.8. Quantitative Real-Time PCR

BMDM cells (2 × 10^5^ per well) were seeded and the cells were stimulated in a 24-well plate as indicated. RNA isolation was carried out by using RNeasy Plus Micro Kit (QIAGEN), according to the manufacturer’s protocol. cDNA was prepared by using the Biozym cDNA Synthesis Kit (Biozym Scientific GmbH, Hessisch Oldendorf, Germany). Quantitative RT-PCR was performed using qPCRBIO Syber Green Mix Hi-ROX (PCR Biosystems, London, UK) and run with the StepOne Real-Time PCR System (Applied Biosystems, Darmstadt, Germany). An initial denaturation step was 2 min at 95 °C, followed by 40 cycles of denaturation at 95 °C for 5 s and amplification at 60 °C for 20 s. All primer pairs used were recently described in [43].

### 4.9. Western Blot Analysis

A total of 2 × 10^6^ cells were stimulated in 2 mL of complete medium in a 6-well plate with plumbagin. Cells were washed with ice-cold PBS. Cell lysis was carried out in 200 μL of 1× RIPA buffer, freshly supplemented with a Phosphatase and Protease-Inhibitor Cocktail (Roche, Mannheim, Germany). Lysates were collected and run on an SDS-PAGE 4–20% gradient polyacrylamide gel (Anamed, Gross-Bieberau, Germany). Proteins were transferred to nitrocellulose membrane via semi-dry Western blot, blocked with 1× Blue Block locking buffer (SERVA, Heidelberg, Germany) for 30 min at RT. Membranes were incubated overnight at 4°C with the primary antibody diluted as suggested by the manufacturer’s protocol. After 1 h incubation with the secondary antibody (HRP-coupled), protein bands were detected by enhanced chemiluminescence (Intas Science Imaging, Göttingen, Germany).

### 4.10. Nuclear Translocation of NFATc1

Bone marrow derived macrophages (BMDMs) from Balb/c mice were seeded onto glass coverslips and incubated in complete media containing M-CSF (30 ng/mL), RANKL (40 ng/mL), with or without plumbagin for 48 h. After incubation, the cells were washed with PBS and fixed with 4% paraformaldehyde and permeabilized with 0.1% Triton X-100 in PBS for 5 min. The cells were blocked with 5% bovine serum albumin (BSA) for 1 h. The cells were incubated with the mouse anti-NFATc1 antibody (BD Biosciences, San Jose, CA, USA), followed by the anti-mouse FITC secondary antibody (Invitrogen, Frankfurt, Germany) for 45 min. Cells were washed and mounted using antifade mounting medium containing DAPI (Abcam, Bristol, UK) and the localisation of NFATc1 was observed under A1R HD25 Nikon confocal microscope under 60× magnification.

### 4.11. ELISA

A total of 2 × 10^6^ cells were stimulated in 2 mL of medium in a cell culture dish with plumbagin and LPS. Supernatants were collected and ELISA measurements were performed in a 96-well plate with BD OptEIA Elisa Kits (BD Biosciences, Heidelberg, Germany) according to the manufacturer’s protocol.

### 4.12. Mitochondrial Extract Preparation

A total of 2 × 10^7^ cells were seeded in 10 mL of complete medium in a 10-cm dish and stimulated for 3 days. For mitochondria extraction, a Mitochondria Isolation Kit (Thermo Scientific, Frankfurt, Germany) with Dounce Homogenization was used following the manufacturer’s protocol.

### 4.13. Mitochondrial Copy Number

A total of 7 × 10^4^ cells were seeded in 1 mL of complete medium in 48 well-plate. On day 4, total genomic DNA was isolated with the innuPREP DNA Mini Kit (Analytik Jena, Jena, Germany). Subsequently, qPCR was run with 2 ng/μL of DNA to amplify and relatively quantify the amount of nuclear and mitochondrial DNA. The mitochondrial copy number in variously stimulated cells was determined by comparing the nuclear DNA amount with the amount of mitochondrial DNA in the cells.

### 4.14. Translation Assay

A total of 1 × 10^6^ cells were seeded in 1 mL of medium in a 24-well plate for 24 h before additional treatment. Cells were stimulated for 6 h. After 6 h of stimulation, the medium was changed to l-methionine free Medium (Gibco DMEM supplemented with 200 µM l-cystine, 2 mM l-glutamine, 1 mM Sodium Pyruvate, 10 mM HEPES) with homopropargylglycine (HPG) according to the Click-iT™ HPG Alexa Fluor™ 488 Protein Synthesis Assay Kit (Thermo Scientific, Darmstadt, Germany) protocol. Cells were incubated for 30 min at 37 °C, 5% CO_2_. Then, the cells were washed with DPBS and fixed with a 4% PFA fixation buffer (BioLegend, London, OK) for 10 min at RT. Cells were washed twice with 3% BSA in PBS and incubated with 200 µL permeabilization buffer (0.5% Saponin) for 10 min at RT. The washing step with DPBS was repeated twice. Cells were then incubated for 30 min protected from light in 250 µL Click-iT reaction cocktail containing Alexa Fluor 488 azide (recipe as prescribed on the kit protocol). The final washing step was carried out with the rinse buffer. The cells were scraped in 400 µL DPBS. FITC fluorescence levels were measured by FACS. Translation was inhibited by adding 10 µM cycloheximide (Santa Cruz Biotechnologies Inc., Dallas, TX, USA).

### 4.15. ROS Measurement

A total of 1 × 10^5^ cells were seeded in 100 µL of medium in white 96-well plates for 48 h. On day 2, the medium was removed. Fifty microlitres (50 μL) Hank’s buffered salt solution (HBSS) for 30 min was added to each well before luminescence measurement. Luminol buffer (10 mg/mL Luminol (Sigma-Aldrich), and HRP 100 U/mL (Sigma-Aldrich) in HBSS) were prepared with and without 60 ng/mL of phorbol 12-myristate 13-acetate (PMA) (Cayman Chemical, Ann Arbor, MI, USA). Fifty microlitres (50 μL) luminol buffer was added to the cells and the luminescence measurement was started as soon as possible upon the addition because of the immediate reaction. The bioluminescence was measured over a time period of around 2 h. To quantify ROS production, the area under the curve was calculated with Graph Pad Prism software (San Diego, CA, USA).

### 4.16. FACS Analysis

For FACS analysis, 1 × 10^6^ cells were used per sample. Cells were blocked for 15 min in PBS, 2% BSA on ice in a total volume of 100 μL. The staining step was carried out on ice for one hour with the appropriate antibody or incubated in the corresponding isotype control diluted as suggested by the manufacturer’s protocol. The surface expression of RANK (R12-31) IgG2a (RTK2758), CD11b (M1/70), CD80 (16-10A1), CD86 (GL-1), F4/80 (BM8) was quantified by flow cytometry by using FACS Canto cytometer (BD Biosciences, Heidelberg, Germany) and BD FACS Diva Software. For the live/dead staining, cells were washed once with DPBS and were stained with SYTOX dye. The cells could be analysed within the FITC channel directly, without further incubation or washing. For measuring mitochondrial activity, the cells were stained with 100 nM MitoTracker (Cell Signaling Technology, Leiden, The Netherlands). After 30 min incubation, the cells were washed with PBS and analysed within the APC-Cy7 channel.

### 4.17. Statistical Analyses

Data are presented as the means ± SD on Graph Pad Prism (San Diego, CA, USA). Comparison between 2 groups was performed as indicated in the figure legend for each experiment.

## Data Availability

The data presented in this study are available within the article.

## Supplementary Materials

The following are available online at https://www.mdpi.com/1422-0067/22/5/2779/s1.
