# Supplementary material for: Plumbagin, a Biomolecule with (Anti)Osteoclastic Properties
Source: Int J Mol Sci. 2021 Mar 9;22(5):2779. doi: 10.3390/ijms22052779 (PMC7967158; doi:10.3390/ijms22052779)
Supplement: Supplementary file 1 [file ijms-22-02779-s001.pdf]

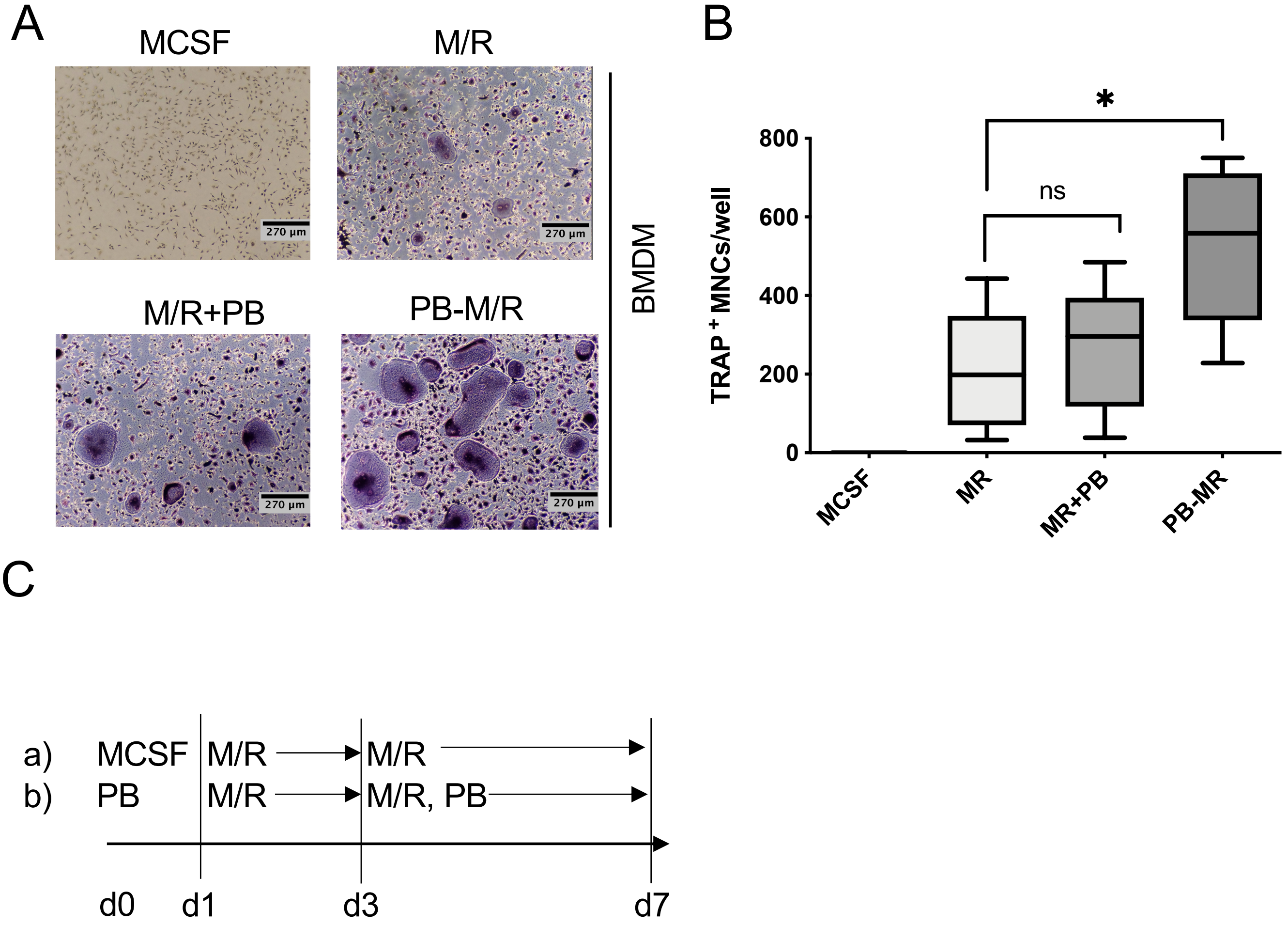

Fig. S1: Plumbagin does not inhibit osteoclast formation. (**a)(b)** BMDM were seeded in a 24 well plate and treated with M-CSF/RANKL or M-CSF/RANKL with 2 µM Plumbagin on day 0 (M/R+PB). In case of plumbagin prestimulation, plumbagin was added for 24 h before addition of M-CSF/RANKL (PB-M/R); **(c)** all other stimulation experiments were performed according to the scheme presented: BMDM were seeded in a 96 or 24 well plate and treated with M-CSF or 2 µM plumbagin on day 0. After 24 hours, M-CSF/RANKL was added to the wells of M/R and PB-MR samples. On day 3, cells were stimulated with M-CSF, M-CSF/RANKL or plumbagin/M-CSF/RANKL, respectively, until the osteoclasts were fully differentiated; **(a)** shows representative pictures (magnification 100X) and **(b)** presents quantification of TRAP stains (n=5).

A

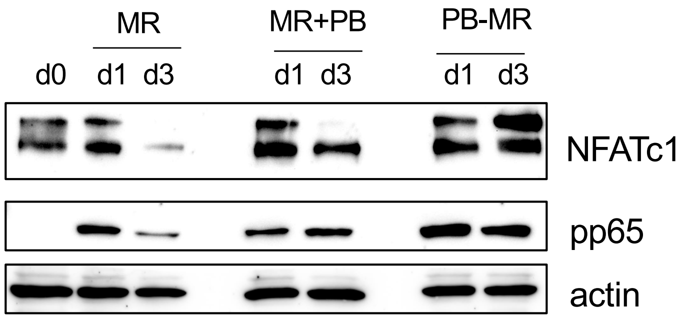

B

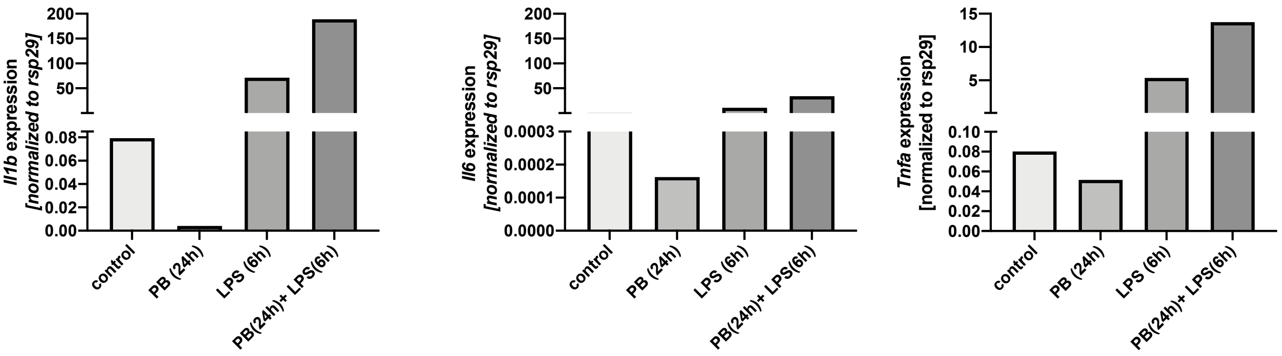

Fig. S2: Plumbagin exaggerates p65-mediated pathways. M-CSF/RANKL and simultaneous or pre-stimulation with plumbagin by using antibodies against NFATc1 and phospho-p65 at day 0, day 1 and day 3. Actin was used as a loading control. The blots are typical examples out of three independent experiments; (**b**) BMDM were incubated with plumbagin for 24 h and subsequently stimulated with 100 ng/ml LPS for 6 hours. Cells treated only with LPS or Plumbagin were used as controls. RT-PCR was performed to determine the expression of the pro-inflammatory cytokines *Il6, Il1 and Tnfa*, respectively.

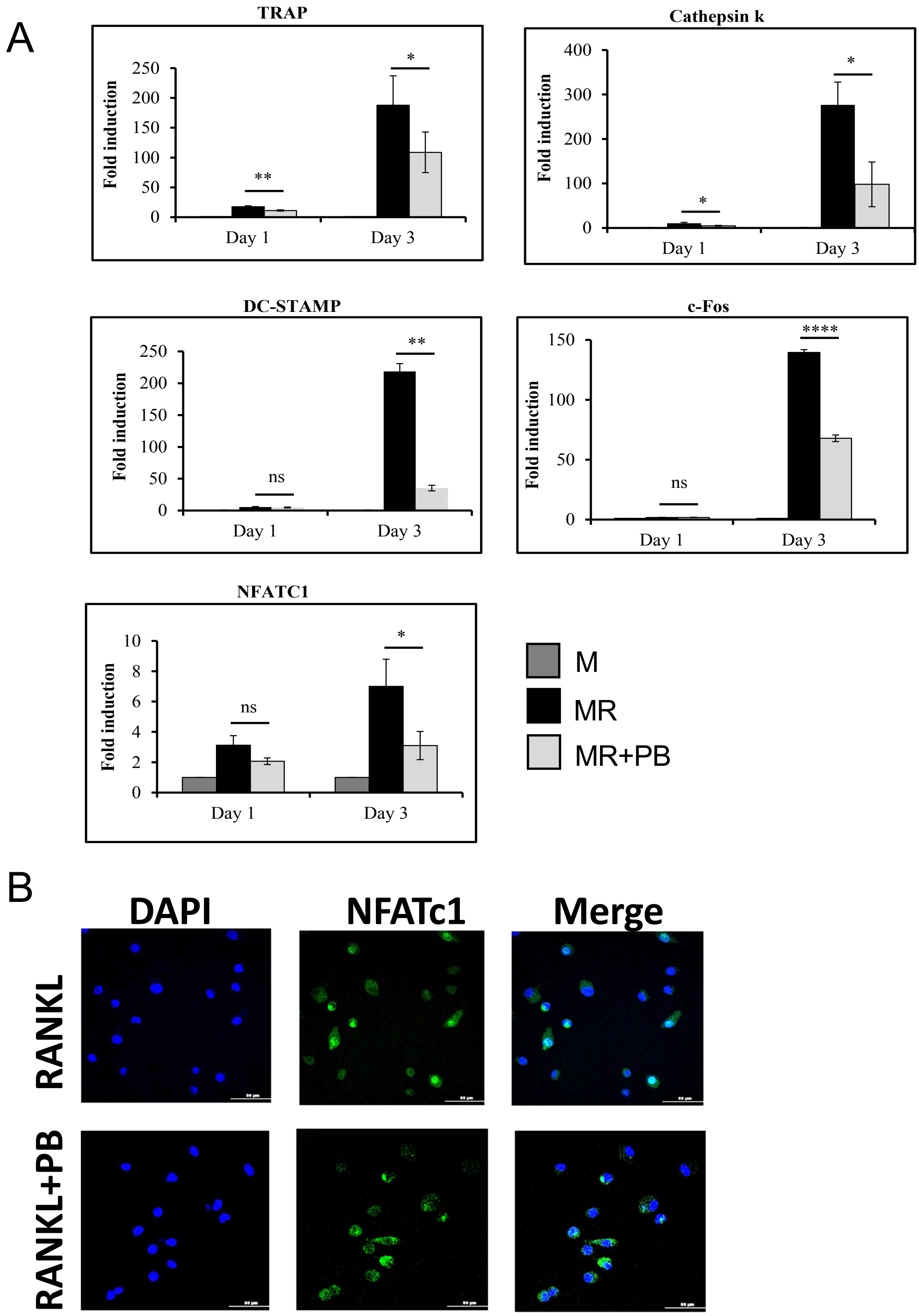

Fig. S3: Plumbagin inhibits the expression of osteoclast-specific genes and NFATc1 nuclear translocation during osteoclast differentiation of BMCs obtained from Balb/c mice. (a) Bone marrow-derived osteoclast precursors were cultured with M-CSF (30 ng/ml) and RANKL (40 ng/ml) in the presence or the absence of plumbagin (2 μM) for 1 and 3 days. The mRNA expression of TRAP, Cathepsin K, DC-STAMP, c-Fos and NFATc1 genes were evaluated using qRT-PCR. Student t-test was applied to infer the statistical significance between the control and experimental group. Data are expressed as mean±SEM. ns-non-significant, *P ≤ 0.05, **P ≤ 0.01, ****P ≤ 0.0001 versus control group; **(b)** RANKL-induced NFATc1 localisation was determined by immunofluorescence microscopy. Briefly, osteoclast precursors were stimulated with RANKL in the presence or absence of plumbagin (2 μM) for 48 hours. The cells were fixed, permeabilized, blocked with BSA and stained for NFATc1 (green) and nuclei (blue). Scale bars show 50 μm, 60X magnification.

A

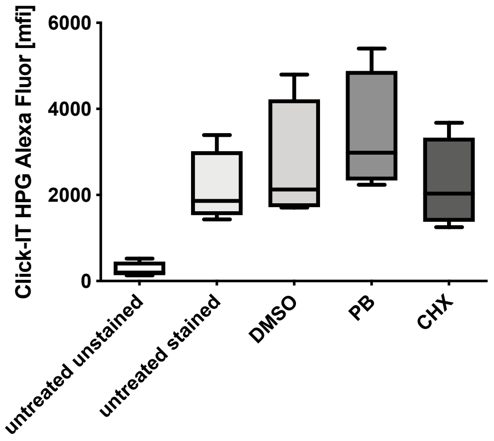

Fig. S4: Plumbagin pre-treatment triggers protein translation. (a) translational activity was measured using a Click-IT assay and subsequent measurement of Alexa488 click-labelled HPG containing proteins. Cells were treated for 6 h as indicated before chasing for 30 min with HPG. Alexa488 fluorescent cells were then measured by FACS analysis. Statistical analysis was performed comparing results to the untreated control using a Friedman test. As a control, cells were pre-treated with 10 µM cycloheximide for 3 h to block protein translation (n=4).
